# Crystal structure of NOD2 and its implications in human disease

**DOI:** 10.1038/ncomms11813

**Published:** 2016-06-10

**Authors:** Sakiko Maekawa, Umeharu Ohto, Takuma Shibata, Kensuke Miyake, Toshiyuki Shimizu

**Affiliations:** 1 Graduate School of Pharmaceutical Sciences, The University of Tokyo, 7-3-1 Hongo, Bunkyo-ku, Tokyo 113-0033, Japan; 2 Department of Microbiology and Immunology, Division of Innate Immunity, The Institute of Medical Science, The University of Tokyo, 4-6-1 Shirokanedai, Minato-ku, Tokyo 108-8639, Japan

## Abstract

Nucleotide-binding oligomerization domain-containing protein 2 (NOD2), a member of the NOD-like receptors family, are crucial for innate immune responses. Mutations of NOD2 have been associated with chronic inflammatory disorders such as Crohn's disease (CD), Blau syndrome (BS) and early-onset sarcoidosis (EOS), but little is known about its signalling mechanism and the role it plays in these diseases. Here, we report the crystal structure of rabbit NOD2 in an ADP-bound state. The structure reveals an inactive closed conformation in which the subdomains in the NOD domain are closely packed by ADP-mediated and inter-domain interactions. Mapping of the BS- or EOS-associated gain-of-function mutations reveals that most of these mutations are located in the NOD subdomain interfaces, and are likely to disrupt the inner domain interactions, facilitating a conformational change to the active form. Conversely, mutations associated with CD are distributed throughout the protein, some of which may affect oligomer formation and ligand binding.

## Abstract (web summary)

NOD2 has a role in host innate immune responses, activating the NF-κB signalling pathway and mutations have been associated with chronic inflammatory disorders. Here, Maekawa *et al*. solved the structure of NOD2 in its inactive form, suggesting a mechanism for autoinhibition.

## Graphical abstract

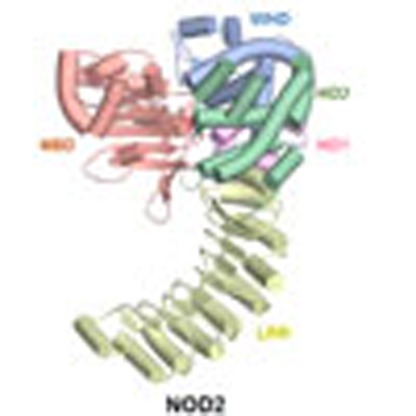

Nucleotide-binding and oligomerization domain (NOD)-like receptors (NLRs), like Toll-like receptors and retinoic acid-inducible gene-I-like receptors, are typical pattern-recognition receptors that recognize pathogen-associated molecular patterns (PAMPs) or damage-associated molecular patterns in the innate immune system^1^,^2^,^3^. While Toll-like receptors are localized to the plasma membrane or endosomes and detect extracellular PAMPs, NLRs act as intracellular receptors for PAMPs^2^,^3^. On activation by ligands, NLRs oligomerize into large multiprotein complexes and mediate innate immune responses, such as the induction of inflammation, autophagy or cell death^2^,^4^.

NLRs belong to the signal transduction ATPases with numerous domains (STAND) clade^5^,^6^ and are composed of three functional regions, an N-terminal effector domain responsible for signal transduction through protein–protein interactions, a central NOD domain with ATPase activity and a C-terminal leucine-rich repeat (LRR) domain presumably involved in ligand recognition. The NOD domain possesses the nucleotide-binding motifs Walker A, Walker B and Sensor 1 (Supplementary Fig. 1)^7^. NLRs are classified into five subfamilies (NLRA, NLRB, NLRC, NLRP and NLRX) according to the type of the N-terminal effector domains they possess, including the caspase-activation and recruitment domain (CARD), pyrin domain and baculovirus inhibitor of apoptosis protein repeat domain^8^.

NOD2, also known as NLRC2 or CARD15, belongs to the NLRC subfamily of NLRs. NOD2 possesses two tandem CARDs that function as an effector domain (Fig. 1a) and is activated by muramyl dipeptide (MDP), a bacterial cell wall fragment^9^,^10^,^11^,^12^. On activation by MDP, NOD2 is believed to undergo self-oligomerization via the central NOD domain, providing a scaffold for recruiting RIPK2 (receptor-interacting serine–threonine kinase 2) through CARD–CARD interactions. Consequently, NOD2 stimulation results in activation of proinflammatory transcription factors including nuclear factor-κB (NF-κB), resulting in increased cytokine expression^13^,^14^,^15^. Recently, NOD2 and the closely related protein NOD1 were shown to interact with the autophagy protein ATG16L1, suggesting that NOD2 plays crucial roles in autophagy^16^.

A considerable number of single-nucleotide polymorphisms (SNPs) have been identified in the NOD2 gene, some of which have been reported to be associated with inflammatory diseases^17^ such as Crohn's disease (CD)^18^,^19^, Blau syndrome (BS)^20^ and early-onset sarcoidosis (EOS)^21^. Some of the CD-associated mutations have been linked to an inability to activate NOD2 (loss-of-function mutations)^11^,^22^, although the precise role NOD2 mutations play in the pathogenesis of CD is not yet established^23^,^24^,^25^,^26^. On the other hand, BS- or EOS-associated mutations have been confirmed to lead to increased basal NF-κB activity (gain-of-function mutations)^21^,^27^.

The proposed mechanism of NLR activation was deduced primarily from activation of the related STAND proteins, Apaf-1 (refs 28, 29, 30) and its homologue CED4 (refs 31, 32), that form apoptosomes. Apaf-1 possesses an N-terminal CARD, a central NOD and C-terminal WD40 repeats as a sensor for cytochrome c. The monomeric inactive form of Apaf-1 undergoes significant structural rearrangement after binding to cytochrome c. This results in the exchange of ADP for ATP in the NOD domain, which enables the formation of ring-like apoptosome structure via self-oligomerization through NOD–NOD interactions^28^,^29^,^30^. Recently, the crystal structure of the inactive form of mouse NLRC4 in an ADP-bound state was determined^33^, and provided detailed insights into the mechanism of auto-inhibition of NLR family proteins. In NLRC4, bound ADP plays an important role in stabilizing the NOD domain in the closed form of NLRC4. Moreover, the LRR domain of NLRC4 masks the surface of the NOD domain, which is necessary for oligomerization^31^, thereby inhibiting oligomerization in the absence of suitable ligands^33^,^34^.

Here, we report the crystal structure of the ADP-bound inactive form of NOD2 with the CARD deleted. Although this compact structure resembled that of the previously determined auto-inhibited structure of NLRC4, the LRR domain was located in a strikingly different position, indicating that the auto-inhibition state of NOD2 was achieved in a different manner, despite the highly conserved structural organization. These results provide structural insight into the regulatory mechanism of NOD2 function and establish the structural basis for understanding the mechanism underlying the effect of pathogenic mutations.

## Results

### Preparation of NOD2 proteins for crystallization

Rabbit (*Oryctolagus cuniculus*, *Oc*) NOD2 (*Oc*NOD2; 86% sequence identity with human NOD2; Supplementary Fig. 1a) was expressed in Sf9 insect cells and was purified as described in the ‘Methods' section. We then used this to engineer a construct lacking the CARD (residues 1–194; *Oc*NOD2ΔCARD). We also generated a construct in which we deleted the loop regions encoded in residues 245–251 and 626–635, in addition to the removal of the CARD (*Oc*NOD2ΔCARDΔloop), because residues 245–251 are not conserved in zebrafish NOD2 (Supplementary Fig. 1a) and residues 626–635 are susceptible to proteolytic cleavage during purification. Moreover, we introduced surface entropy reduction (SER) mutations (E943A, E944A, K635A, E636A, E709A and K711A) into the *Oc*NOD2ΔCARD construct to produce the *Oc*NOD2ΔCARD^SER^ construct. These CARD-defective NOD2 proteins for structural study would be inactive, because the CARD domain is required for triggering NF-κB activation through the serine–threonine kinase RIPK2.

The *Oc*NOD2Δloop construct exhibited activity comparable to the wild-type *Oc*NOD2 in response to MDP, while the *Oc*NOD2^SER^ construct exhibited no activity in NF-κB reporter assays in HEK293T cells (Supplementary Fig. 2). Gel filtration chromatography analyses revealed that full-length *Oc*NOD2, *Oc*NOD2ΔCARD, *Oc*NOD2ΔCARDΔloop and *Oc*NOD2ΔCARD^SER^ exist as monomers in solution (Supplementary Fig. 3).

### Crystal structure of NOD2 in the ADP-bound form

**Figure 1:**
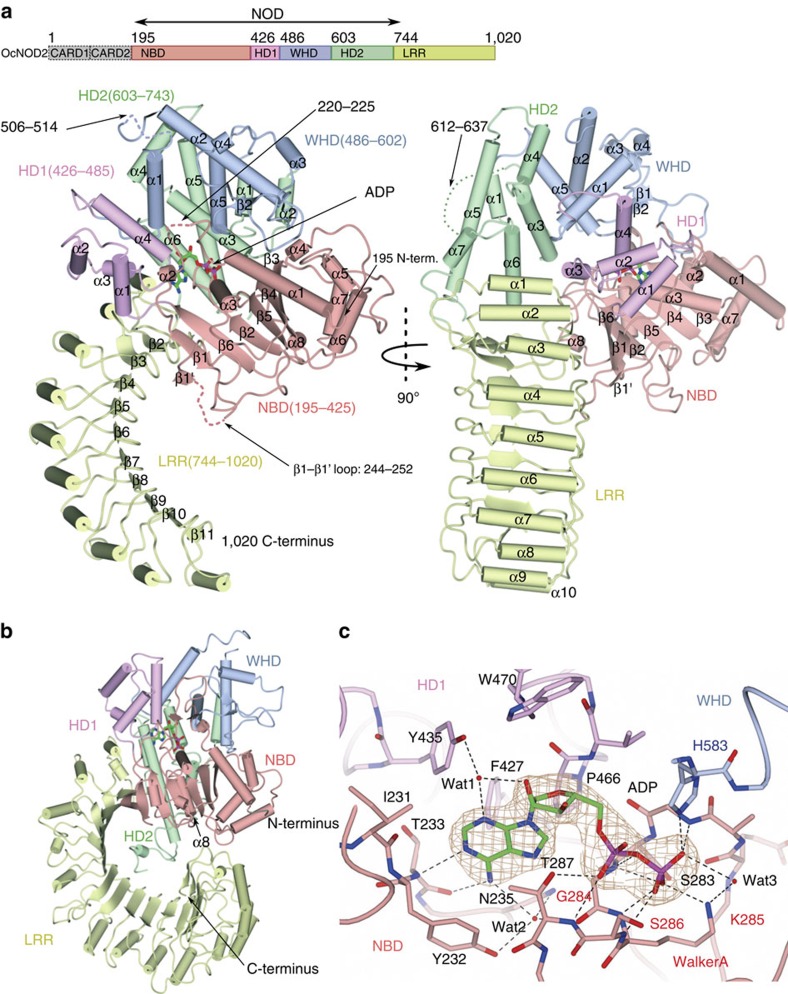
Structure of *Oc*NOD2. (**a**) Structure of *Oc*NOD2ΔCARDΔloop (Form 2). The domain organization of *Oc*NOD2 is shown at the top. Front view (left) and side views rotated by 90° along a vertical axis relative to the left (right). *Oc*NOD2 is comprised of five sequential domains: NBD (195–425, pink), HD1 (426–485, purple), WHD (486–602, cyan), HD2 (603–743, light green) and LRR (744–1,020, yellow). The secondary structures are labelled. The ADP molecule is shown as a stick structure with the C, O, N and P atoms coloured green, red, blue and magenta, respectively. The regions missing in the refined model are indicated by dashed lines. The colouring scheme for each domain is used throughout all of the figures. (**b**) The structure of mouse NLRC4 (PDB code 4KXF) displayed in nearly the same orientation as *Oc*NOD2 ((**a**) left). (**c**) Detailed view of the ADP-binding site of *Oc*NOD2. Residues involved in the recognition of ADP are shown as a stick structure and labelled. The 2*F*o−*F*c electron-density map is shown in brown mesh around ADP. Hydrogen bonds are indicated by dashed lines. Red spheres represent water molecules.

We crystallized *Oc*NOD2ΔCARDΔloop in two forms (form 1 and form 2) under different crystallization conditions and determined the crystal structures at 2.3 Å (form 1) and 3.3 Å (form 2) resolutions (Fig. 1, Supplementary Fig. 4a,b, Table 1). In addition, we determined the structure of *Oc*NOD2ΔCARD^SER^, which is isomorphous to the form 1 of *Oc*NOD2ΔCARDΔloop crystal, at 3.1 Å resolution (Supplementary Fig. 4a, Table 1). The asymmetric unit of the form 1 and form 2 crystals contained one and two NOD2 molecules, respectively. The structures of the NOD2 molecules in these crystals were essentially the same, with root mean squared deviations (r.m.s.d.) between 0.4 and 1.1 Å (Supplementary Fig. 4a). Since we observed no noticeable differences among these three crystal structures, we refer, hereafter, to the structure of *Oc*NOD2ΔCARDΔloop in the form 2 crystal unless otherwise noted.

The crystal structure showed that ADP-bound NOD2 folded into a hook-shaped structure with dimensions of ∼70 × 80 × 40 Å, consisting of a NOD domain (residues 195–744) and a LRR domain (residues 745–1,020; Fig. 1a). The NOD domain can be further divided into the nucleotide-binding domain (NBD, residues 195–425), helical domain 1 (HD1, residues 426–485), the winged-helix domain (WHD, residues 486–602) and helical domain 2 (HD2, residues 603–743), similar to other NOD domains with known structures (Fig. 1a,b)^28^,^31^,^32^. The NBD, HD1 and WHD subdomains superimposed well with the corresponding subdomains of NLRC4, with r.m.s.d. values of 1.9, 1.6 and 1.8 Å, respectively (Supplementary Fig. 5a). HD2, the functional role of which is not well characterized, adopted a different tertiary structure with a large r.m.s.d. value of 3.1 Å (Supplementary Fig. 5b), which is consistent with the fact that HD2 is less homologous to the other NOD subdomains^35^. The LRR domain of NOD2 consisted of ten LRR units and formed a typical horseshoe-like structure in a single curvature, with α-helices forming the convex surface and β-strands in the concave faces. Several loop regions (residues 220–225, 244–252, 506–514, 613–636 and 703–714) are not included in the model due to the poor electron-density. These regions contain the loops that were deleted for crystallization (β1–β1' loop in NBD and α1–α2 loop in HD2; Fig. 1a, Supplementary Fig. 4a).

### ADP is critical for maintaining NOD2 in a closed form

In the crystal structure, the electron density of the ADP molecule was observed clearly, even though it was not supplemented during purification and crystallization (Fig. 1c). ADP embedded at the centre of the NOD domain bridged the NBD and HD1, and bound to the WHD (Fig. 1a,c), allowing the subdomains to be closely packed. As the resulting structure of NOD2 folded into a compact tertiary arrangement, we concluded that the crystal structure of *Oc*NOD2ΔCARD is that of a closed and inactive conformation.

The main- and side-chain atoms from the Walker A motif (GKS, residues 284–286) and its surrounding region (residues 282–287) formed five hydrogen bonds with the β-phosphate and three with the α-phosphate groups of ADP (Fig. 1c). The well-ordered water (Wat3) was coordinated by the β-phosphate of ADP and K285. The adenosine moiety of ADP was buried in the cavity formed between the NBD and HD1 and surrounded by the residues I231, Y232, T233, N235, T287, F427, Y435, P466 and W470 (Fig. 1c). The main-chain atoms of T233 formed hydrogen bonds with the N1 and N6 atoms of the adenine base (Fig. 1c). The side-chain atom of Y435 formed water (Wat1)-mediated hydrogen bonds with the N3 atom of the adenine base and the 2′-hydroxyl group of the ribose moiety (Fig. 1c). Similarly, Y232, N235 and G284 interacted indirectly with the N6 atom of the adenine base via water (Wat2)-mediated hydrogen bonds. The conserved H583 residue in the WHD coordinated the β-phosphate of ADP via hydrogen bond formation with a distance of 2.8 Å (Fig. 1c, Supplementary Fig. 1b). This His-mediated interaction with ADP was shown to be important for auto-inhibition in NLRC4 (ref. 33). ADP in the *Oc*NOD2 structure was coordinated in a manner similar to that of the structures of other NOD-containing proteins, including NLRC4 and Apaf-1 (Supplementary Fig. 6)^28^,^29^,^33^.

### Subdomain interfaces of NOD domain

In addition to the ADP-mediated interactions, the closed and inactive form of NOD2 was stabilized by subdomain interactions. The NBD formed inter-domain interactions with HD1 over a contact area of 439 Å^2^. The α2-helix and the flanking loop of the NBD made contact with the α1 helix of HD1 and Walker A interacted with the α4 helix of HD1 (Fig. 1a).

**Figure 2:**
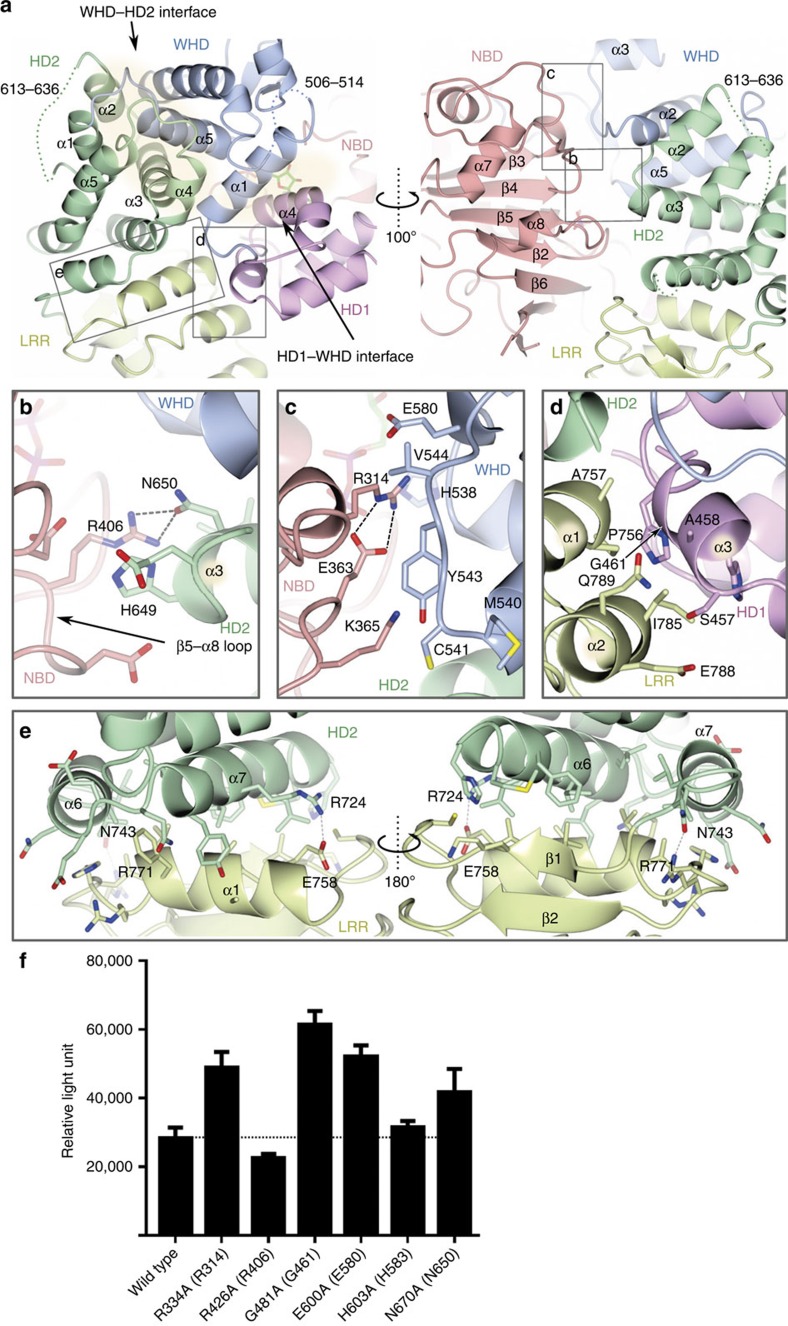
Inter-domain interactions in the NOD domain. (**a**) Inter-domain interactions in the NOD domain viewed from two different orientations. The WHD–HD2 and HD1–WHD interfaces are highlighted by semi-transparent beige ovals. Structural elements involved in the interactions are labelled. (**b**–**e**) Magnified views of the NBD–HD2 (**b**), NBD–WHD (**c**), LRR–HD1 (**d**) and LRR-HD2 (**e**) interfaces. Each panel corresponds to the region indicated in **a**. Hydrogen bonds and salt bridges are shown as dashed grey lines. (**f**) Constitutive NF-κB activation by human NOD2 mutants associated with auto-inhibition by luciferase reporter assay using HEK293T cells. Residues in parenthesis are derived from *Oc*NOD2. Data represent the mean relative light units (RLU) of NF-κB activity in the absence of MDP (*n*=6, ±s.d.). Dotted lines indicate activity of wild-type NOD2.

The interface between the NBD and HD2 was relatively small, with a contact area of only 223 Å^2^ (Fig. 2a,b). Of note, R406 in the Sensor 1 motif (TSR, residues 404–406) of the NBD formed a hydrogen bond with N650 in HD2 (Fig. 2b). The corresponding Arg in the ATP-bound active form of CED4 is known to coordinate the γ-phosphate group of ATP^32^; thus, R406 of NOD2 possibly interacts with ATP in the active form, suggesting that this interaction would be important for stabilizing the closed conformation. Correspondingly, the N670K mutation (N670 is the human counterpart of N650 in *Oc*NOD2) results in constitutive activation of NOD2 (refs 21, 36). In NLRC4, R406 in *Oc*NOD2 is not conserved (Supplementary Fig. 1c). In addition, HD2 domain itself and its relative orientation to the other domains of NOD domain were quite different between NOD2 and NLRC4 (Fig. 1, Supplementary Fig. 5). Therefore, interaction between NBD and HD2 via R406 and N650 is unique to NOD2.

The NBD also interacted with the WHD over a contact area of 373 Å^2^. R314 interacted electrostatically with E363 in the Walker B motif (LLTFDGFDEFK, residues 355–365) and simultaneously with E580 of the WHD (Fig. 2a,c). These residues are conserved across NOD2 of all species (Supplementary Fig. 1). In NLRC4, R206 (counterpart of R314 in *Oc*NOD2) interacted with E252 in the Walker B motif (counterpart of E363 in *Oc*NOD2) and D407 (Supplementary Fig. 1c). D407 was not a counterpart of E580 of *Oc*NOD2 but occupied the similar position, establishing the similar NBD–WHD interaction as NOD2 (ref. 33). Consistent with this, the R334Q, R334W, E383G and E383K mutations (R334 and E383 are the human counterparts of R314 and E363 in *Oc*NOD2, respectively) result in constitutive activation of NOD2 (refs 20, 21, 22, 36), demonstrating the importance of these interactions for keeping NOD2 in an inactive conformation. E600A mutation (E600 is the human counterpart of E580 in *Oc*NOD2) also resulted in constitutive activation described below.

The WHD formed inter-domain interactions with HD1 and HD2 as well as the NBD. The α1 helix in the WHD was central to the interface between HD1 and the WHD (with a contact area of 696 Å^2^), forming hydrophobic interactions with the α4 helix in HD1 (Fig. 2a). Interactions between the WHD and HD2 were mediated by extensive hydrophobic interactions over a contact area of 1,419 Å^2^ (Fig. 2a). The α5 helix in the WHD is positioned on the groove formed by the α1–α5 helices. The α4 helix and the surrounding region in the WHD are highly conserved among different species of NOD2 and contain the conserved residue H583, which coordinated the β-phosphate of ADP (Supplementary Fig. 1), indicating the importance of this interface for stabilizing the closed form described above.

### Interface between NOD and LRR domains

The LRR domain of NOD2 formed inter-domain interactions with the NOD domain, and these could be subdivided into interactions with the HD2 and HD1 interfaces over contact areas of 865 and 319 Å^2^, respectively (Fig. 2a,d,e). In the LRR–HD1 interface, the α3 helix (HD1) was packed against the α1 and α2 helices of the LRR, mainly through van der Waals contacts (Fig. 2d). Small residues in the α3 helix (HD1), including G461 and S457, established shape complementarity at the interface. LRR–HD1 interaction pulled the HD1 toward the LRR in NOD2, while no interaction was observed in the crystal structure of NLRC4 (Fig. 2a,d)^33^. The LRR domain of NOD2 interacted intimately with HD2 via the formation of extensive hydrophobic interactions between the first LRR unit of the LRR domain and the α6 and α7 helices of HD2 (Fig. 2e). As a result, HD2 acted as a lid on the N-terminal side of the LRR domain to protect the hydrophobic core of the LRR from exposure to the solvent. Formation of hydrogen bonds between R771 (LRR) and N743 (HD2) and a salt bridge between E758 (LRR) and R724 (HD2) further support the interactions at both sides of the α1 helix of LRR (Fig. 2e). Consistent with our structural analysis, truncation of LRR domain results in enhanced NF-κB activation^27^,^37^, probably because it disrupts the close packing of HD1/HD2 and LRR. Supportively, G481D mutation in HD1 (G461 in *Oc*NOD2), which interacts with LRR, causes auto-activation of NOD2 (ref. 36). In NLRC4, the concave surface of the LRR domain interacts with several α-helices (HD2) and a β-hairpin (NBD) protruding from the NOD domain, especially at phosphorylated S533 (HD2; Fig. 1b), locking the closed structure^33^. By contrast, the concave surface of the LRR domain in NOD2 made little contact with the NBD, with a contact area of only 294 Å^2^. The concave surface of LRR in NOD2 was almost unoccupied, although the β1–β1′ loop (NBD) of NOD2 corresponding to the β-hairpin in NLRC4 could extend to the surface of the LRR (Fig. 1a, Supplementary Fig. 4).

### Functional analysis for mutants related with auto-inhibition

Our structural analysis revealed that the closed and inactive form of NOD2 was achieved by subdomain interactions and ADP-mediated interactions. Several groups have already performed the reporter assay for the constitutive activity of SNP-based NOD2 mutants^22^,^27^,^36^. We also performed the NF-κB-dependent luciferase reporter assay using HEK293T cells. To prove the contribution of an individual amino acid and to minimize the side effect of the mutation, we mutated the following residues to Ala: R334A (R314 in *Oc*NOD2; NBD–WHD), R426A (R406 in *Oc*NOD2; NBD–HD2), G481A (G461 in *Oc*NOD2; HD1–LRR), E600A (E580 in *Oc*NOD2; NBD–WHD), H603A (H583 in *Oc*NOD2; WHD–ADP), N670A (N650 in *Oc*NOD2; NBD–HD2). Expectedly, the R334A, G481A, E600A and N670A mutants exhibited higher level of constitutive activity than wild-type protein (Fig. 2f). On the other hand, R426A and H603A mutants did not, probably because Ala substitution was insufficient to abolish the interaction.

### Potential ligand-binding site on the LRR domain

**Figure 3:**
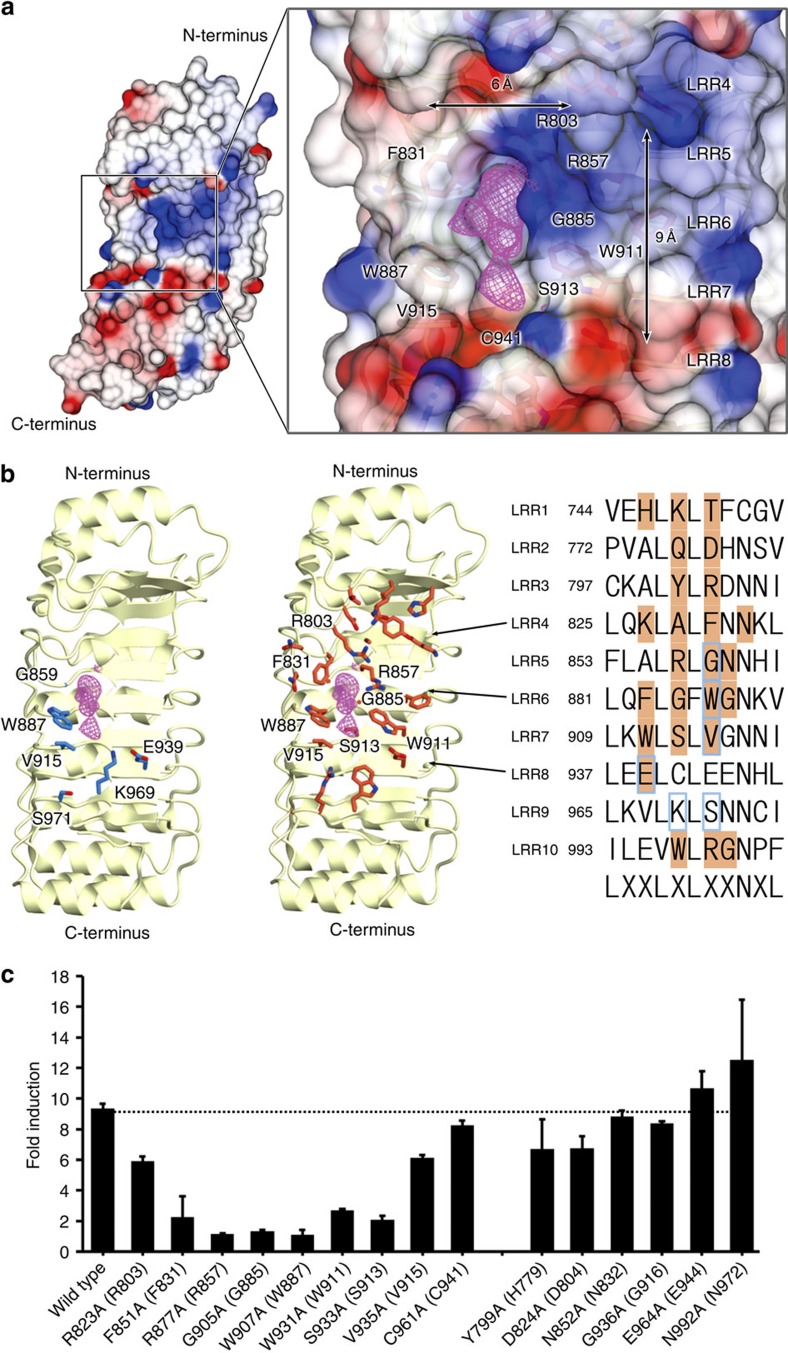
Potential ligand-binding site on the concave surface of LRR. (**a**) Semi-transparent surface representation showing the concave face of the LRR domain. The potential ligand-binding site is indicated by a rectangle (left) and is magnified on the right side of the panel. The residues forming the pocket are shown as stick structures and labelled. Positive and negative electrostatic potentials are shown in blue and red, respectively. The *F*o−*F*c difference electron density in the pocket is contoured at the 3.0 sigma level with magenta mesh. (**b**) The residues in the ligand binding implicated by mutational study^27^ (left) and sequence conservation analysis (middle) are shown as blue and coral stick structures, respectively. The residues conserved among all species of NOD2 (listed in Supplementary Fig. 1) are shown in the middle panel, except for those at positions 1, 4, 6, 9 and 11, of the first 11 residues of each LRR (shown in the right panel). The residues shown in the left and middle panels are highlighted in the right panel in blue and coral, respectively. (**c**) MDP induced NF-κB activation of the human NOD2 mutants for the residues forming the proposed MDP-binding pocket and its peripheral region. Residues in parenthesis are derived from rabbit NOD2. Luciferase activity was measured by NF-κB-dependent luciferase reporter assay using HEK293T cells co-expressing rabbit NOD2 and human SLC15A3. Data represent the mean fold induction of NF-κB activity (*n*=3, ±s.d.), calculated as the relative light units (RLU) of cells stimulated with MDP divided by the RLU of non-stimulated cells. Dotted lines indicate activity of wild-type NOD2.

Generally, the LRR domain of NLRs is believed to function as a pathogen sensor^7^. Previous mutational studies of NOD2 have proposed that the potential ligand-binding site lies on the concave surface of the LRR domain^27^. Accordingly, we found a hydrophobic pocket on the concave surface of the LRR domain with dimensions of ∼9 × 6 × 6 Å, suitable for accommodating glycan or peptide moieties of MDP (Fig. 3a). R803, F831, R857, W887, W911, V915 and C941 formed the inner wall of the pocket and G885 and S913 formed the floor. These residues are highly conserved among different species of NOD2, and W887 and V915 were implicated previously in ligand binding^27^, strongly suggesting that this pocket is the ligand-binding site (Fig. 3b). Interestingly, the pocket was occupied by an unknown electron density that could not be assigned to the molecules used for purification and crystallization (Fig. 3a). To confirm the functional importance of this pocket, we performed the NF-κB-dependent luciferase reporter assay of the NOD2 mutants of the residues forming the putative MDP-binding pocket (R803, F831, R857, G885, W887, W911, S913, V915 and C941 in *Oc*NOD2). Expectedly, most of the mutants except R803, V915 and C941 reduced or lost the responsiveness to MDP stimulation, supporting our structural suggestion (Fig. 3c). Ala substitution in R803, V915 and C941 would be insufficient to abolish the MDP binding. On the other hand, the Ala mutants of residues that were located in the peripheral region of the pocket (H779, D804, N832, G916, E944 and N972 in *Oc*NOD2) showed the same level of MDP response as wild-type protein.

## Discussion

**Figure 4:**
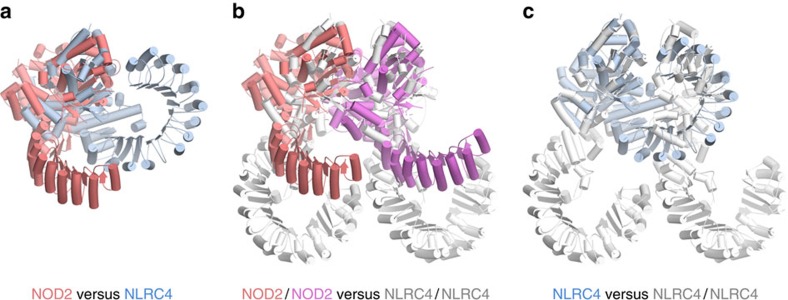
Orientation of the LRR domain. (**a**–**c**) Superpositions of *Oc*NOD2ΔCARD and inactive mNLRC4ΔCARD (**a**), *Oc*NOD2ΔCARD and NLRC4/NLRC4 lateral dimers of NLRC4–NAIP2 inflammasome (**b**), and inactive mNLRC4ΔCARD and NLRC4/NLRC4 lateral dimers of NLRC4–NAIP2 inflammasome (**c**) using the NBD–HD1 regions for alignment (residues 195–485 and 95–355 for NOD2 and NLRC4, respectively). *Oc*NOD2ΔCARD, inactive mNLRC4ΔCARD and NLRC4 lateral dimers of NLRC4–NAIP2 inflammasome are shown in red/pink, light blue and grey, respectively.

Here, we determined the crystal structure of the inactive ADP-bound form of NOD2, and revealed that ADP-bound NOD2 is maintained in an auto-inhibited/closed form through ADP-mediated and inner domain interactions of the NOD domain. Although NOD2 shares the common ADP-mediated auto-inhibition mechanism observed in NLRC4 and Apaf-1 (refs 28, 29, 33), our structural work provides new insights into the mechanism of NLR auto-inhibition (Fig. 4).

In the auto-inhibited form of Apaf-1, the caspase-9-binding surface of the CARD is partially buried, making it unavailable for recruitment of caspase-9, and the WD40 repeats further stabilize this auto-inhibited conformation. The closed form changes to the open form on ligand binding, with rotation of the WHD–HD2 modules against the NBD–HD1 modules^28^,^29^,^30^. As the similar structural reorganization was also observed in NLRC4 (refs 38, 39), NOD2 would undergo a similar structural remodelling during activation with WHD–HD2–LRR rotation as a rigid body relative to the NBD–HD1 modules. Of note, the WD40 domain is positioned to sterically occlude an adjacent Apaf-1 molecule and consequently sequester Apaf-1 in a monomeric state. In NLRC4, crystallographic analysis showed that the LRR domain locks the closed form by spatially bridging the NBD and HD2, achieving auto-inhibition with the assistance of ADP^33^. Similar to Apaf-1, superpositioning of inactive NLRC4 with a lateral dimer of the active NLRC4–NAIP2 inflammasome shows that the LRR domain of NLRC4 heavily overlaps with the protomer of the lateral dimer, suggesting that the LRR domain of NLRC4 inhibits the putative NOD-mediated assembly by steric hindrance (Fig. 4c)^33^,^34^. In addition, the HD2–LRR interaction in NLRC4 locks the molecule into the auto-inhibited form. By contrast, no severe steric clash would occur in the assembly of NOD2 when we superposed two molecules of *Oc*NOD2 with both protomers of a lateral NLRC4 dimers of NLRC4–NAIP2 inflammasome structure^38^,^39^ and a lateral CED4 dimers of octameric CED4 structure^32^ (Fig. 4b, Supplementary Fig. 7), because the LRR domain in NOD2 was oriented outward against the NBD–HD1 regions by 30° compared with NLRC4 (Fig. 4a). In addition, the concave surface of the LRR domain was not occupied (Fig. 4b). Therefore, the auto-inhibition mechanism by which the LRR domain functions as a repressor of NLR activation by masking the interface necessary for NOD–NOD interaction^33^ may not be fully applicable neither for NOD2/NOD1, a closely related orthologue of NOD2 (ref. 10), nor for some other members of the NLR family. Thus, despite a highly conserved structural organization, the mechanism of auto-inhibition in NOD2 was achieved via a different mechanism.

**Figure 5:**
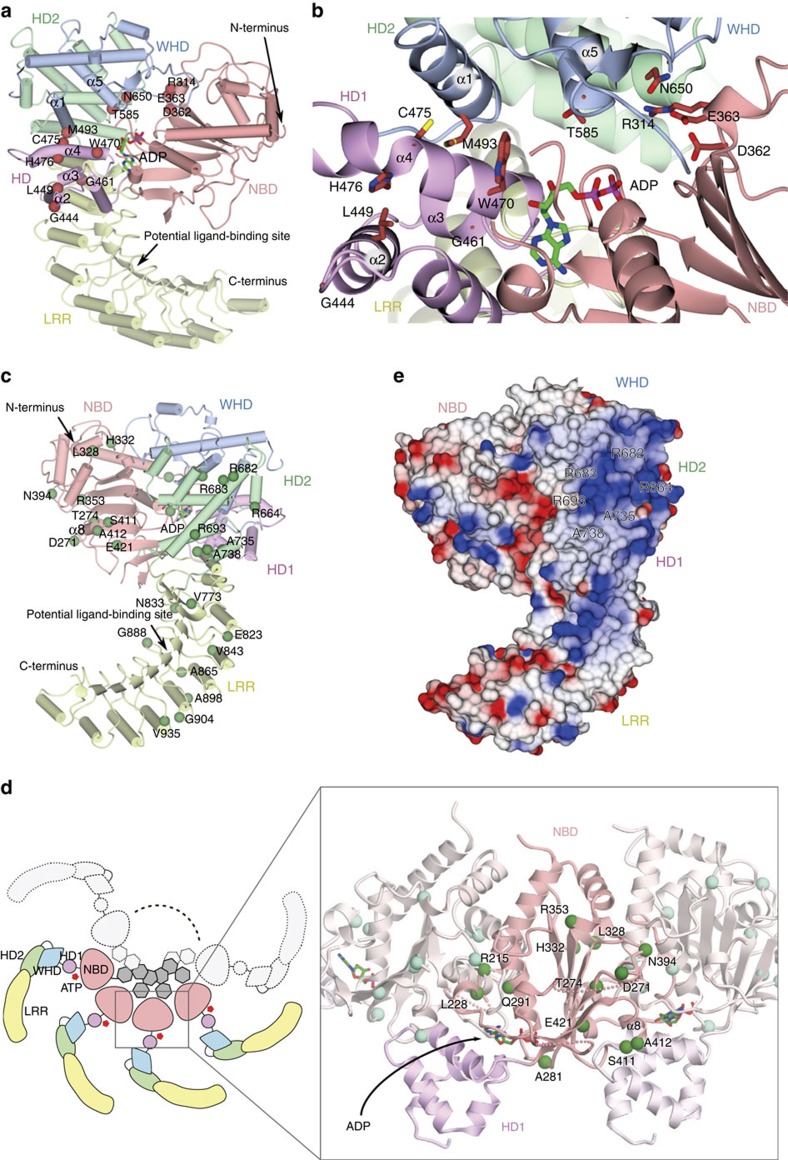
Disease-related mutations. (**a**) Mapping of the BS/EOS-associated mutations in the structure of *Oc*NOD2. Mutated residues are shown as coral spheres. (**b**) Detailed view of **a**. Mutated residues are shown as coral sticks. (**c**) Mapping of CD-associated mutations in the structure of *Oc*NOD2 rotated 180° from the orientation shown in **a**. Mutated residues are shown as green spheres. (**d**) Putative oligomer model of NOD2. The oligomer model shown in cartoon (left). Magnified view of the NBD–HD1 region in the three adjacent protomers of NOD2 (right). The oligomer model is constructed by superimposing the *Oc*NOD2 structure onto the CED4 octamers (PDB code 3LQQ) using the NBD domain as reference. The middle protomer is coloured in pink (NBD) and purple (HD1) and the rest of the protomers are shown in light colours. CD-related mutations in the NBD domain are shown as green spheres in the middle protomer and as palegreen spheres in the adjacent protomers. (**e**) Electrostatic surface potential of *Oc*NOD2 shown in the same orientation as in **c**. CD-related mutations located on the positively charged surface of HD2 are labelled.

Because the potential ligand-binding site on the concave surface of the LRR is distantly positioned from the NOD domain (Fig. 5a), we cannot predict how ligand binding affects the structure of NOD2. It is possible that ligand binding allosterically alters the auto-inhibitory inter-domain interactions. Moreover, the exchange of ADP for ATP would induce structural changes in the NOD domain that could allow NOD2 activation. Further studies are needed to clarify how ligand binding leads to the oligomerization of NOD2.

Many SNPs in the NOD2 gene are associated with human inflammatory disorders^17^,^18^,^19^,^20^,^21^. Gain-of-function mutations, which increase the basal activity of NF-κB, have been associated with the pathogenesis of BS or EOS^20^,^21^, whereas some loss-of-function mutations have been correlated with susceptibility to CD^18^,^19^. To study how these SNPs affect NOD2 structure and function, we mapped SNPs listed in the UniProt database^40^ to the structure of NOD2 (Fig. 5, Supplementary Table 1). The numbering schemes of the human SNPs described below have been converted to rabbit NOD2 residue numbers.

Most of the gain-of-function mutations (a total of 14 mutations) that lead to BS or EOS are concentrated on or near the domain interfaces of NBD, HD1, WHD and HD2 in the NOD domain; three mutations exist in the NBD, seven in HD1, three in the WHD and one in HD2 (Fig. 5a,b, Supplementary Table 1). No mutations mapped to the LRR domain. Mutation of these residues would destabilize the closed form of NOD2 and facilitate the conformational change from the inactive to the active form, resulting in constitutive activation of the protein.

Mutation of G444 and L449 in the α2 helix and H476 in the α4 helix of HD1 (G444W, L449F and H476L) would attenuate HD1–WHD, HD1–NBD and HD1–ADP interactions, possibly by impairing the correct folding of HD1 (Fig. 5b). G461 in the α3 helix (HD1) fits snugly into the surface of the LRR domain; thus, G461D mutation would disrupt the interaction between HD1 and the LRR domain. W470 and C475 in the α4 helix (HD1) and M493 in the α1 helix of the WHD are directly involved in the hydrophobic interaction with HD1 (Fig. 5b), and mutation of these residues (W470L, C475Y and M493T) would weaken this interaction. R314 in the NBD forms a hydrogen bond with E363 and interacts electrostatically with E580 of the WHD (Figs 2c and 5b). Thus, mutation of these residues (R315Q, R314W and E363G, E363K) would disrupt these interactions and weaken the association between the NBD and WHD. In addition, the Walker B motif residues D362 and E363 are believed to be involved in the hydrolysis of ATP^6^, and it has been suggested that the constitutive activity conferred by mutation of these residues (D362E, E363G and E363K) may be attributed to of the inability of these mutants to hydrolyse ATP^41^. T585 in the WHD forms a hydrogen bond with the conserved residue H583, effectively stabilizing the side chain of H583 for coordinating the β-phosphate group of ADP (Fig. 5b). Thus, the T585N mutation would influence this interaction. Finally, N650 (HD2) forms a hydrogen bond with the Sensor 1 motif residue R406 in the NBD (Figs 2b and 5b), and mutation of this residue (N650K) would result in disruption of the hydrogen bond, attenuating the NBD–HD2 interaction.

In contrast to the concentrated distribution of mutations associated with BS or EOS, mutations associated with CD (a total of 31 mutations) were scattered throughout the NOD2 structure in all domains except HD1; 13 mutations mapped to the NBD, one to the WHD, seven to HD2 and ten to the LRR domain (Supplementary Table 1, Fig. 5c). Approximately two-thirds of the mutations exist on the surface of the NOD2 structure. In the putative oligomer model of NOD2 based on the octameric assembly of CED4, most of the mutations in the NBD are located near the self-oligomerization interface^32^ (Fig. 5d). The interfaces between protomers are essentially the same as the oligomeric model based on the NLRC4–NAIP2 inflammasome structure^38^,^39^ (Supplementary Fig. 7). In this model, R215, L228, Q291, L328 and H332 lie adjacent to one neighbouring protomer and D271, T274, N394, S411, A412 and E421 lie adjacent to the other protomer (Fig. 5d). In particular, S411 and A412 are located near the α8 helix in the NBD, which is important for the oligomerization of STAND family proteins^32^,^33^,^42^. Thus, mutation of any of these residues may impair the oligomerization of NOD2, leading to a loss of function. Mutations in HD2 (R664W, R682W, R683C, R693C, A705G, A735V and A738V) are not involved in interaction with other NOD subdomains, but these residues form a positively charged surface (Fig. 5e) that may be involved in association with the membrane or interaction with other proteins. Of note, several mutated residues for SER mapped to HD2. Mutation of these residues may alter the charge distribution, resulting in reduction of the activity of *Oc*NOD2ΔCARD^SER^. The NOD2 L1007finsC polymorphism, which is most prevalent in CD patients, results in a frameshift mutation that generates a truncated NOD2 protein^18^,^19^. It was reported that L1007fsinsC mutant failed to localize to the plasma membrane^43^. Several proteins such as erbin^44^, FRMPD2 (ref. 45) and ATG16L1 (ref. 16) are implicated in NOD2 recruitment to plasma membrane. It is possible that the most C-terminal region of NOD2 mediates membrane association directly or indirectly through other proteins. NOD2 localization to the plasma membrane may encourage more efficient interaction with its ligand^46^. Thus, the loss of responsiveness to MDP of L1007fsinsC mutant may be attributable to the loss of interaction to the plasma membrane. In addition, as this mutant truncates LRR10, which forms a peripheral region of the ligand pocket, it might be unable to form the responsive ligand pocket. Moreover, it remains possible that the LRR domain is also involved in ligand-induced NOD2 oligomerization demonstrated in the NAIP2–NLRC4 inflammasome structure, in which the LRR domain contributes to NLRC4 oligomerization via charge–charge interaction between two LRR domains of an NLRC4 lateral dimer.

Mutations in LRR are spread throughout the domain. Notably, the G888R mutation is located next to W887, which is a constituent of the proposed MDP-binding pocket (Figs 3 and 5c). Introduction of a larger Arg side chain may cause local structural changes to the pocket, leading to inability of the mutant protein to respond to MDP. The effects of the remainder of the mutations are undefined, but could relate to ligand recognition, stability of the protein, modulation of LRR conformation and interaction with other proteins.

This structural study provides a better understanding of the regulatory features of NOD2 and the mechanisms by which reported mutations result in the development of diseases at a molecular level. These insights provide a structural platform for the development of improved therapeutic intervention targeted to NOD2.

## Methods

### Preparation of recombinant proteins

The gene encoding full-length rabbit NOD2 (*Oc*NOD2; residues 1–1,020), *Oc*NOD2 (residues 195–1,020; *Oc*NOD2ΔCARD), *Oc*NOD2ΔCARD with residues 245–251 and 626–635 deleted (*Oc*NOD2ΔCARDΔloop) and *Oc*NOD2ΔCARD with SER mutations (K635A, E636E, E709A, K711A, E943A and E944A; *Oc*NOD2ΔCARD^SER^) were cloned between the *Eco*RI and *Spe*I sites of the modified pFastBac Dual vector (Life Technologies). Recombinant proteins were expressed in Sf9 cells from *Spodoptera frugiperda* (Thermo Fisher Scientific Inc.) with an N-terminal hexahistidine-FLAG tag followed by PreScission Protease recognition sequences. Sf9 cells were infected with recombinant baculoviruses and incubated at 27 °C for 72 h. After the incubation, cells were collected, re-supspended and lysed by sonication in a buffer containing 25 mM Tris pH 8.0, 500 mM NaCl, 10% glycerol and 1 mM DTT. The proteins were purified from cell lysate by Ni-NTA resin (QIAGEN) and Superdex 200 gel filtration column (GE Healthcare). After the tag removal by PreScission Protease, the proteins were further purified by HiTrap Q anion-exchange column (GE Healthcare) and Superdex 200 gel filtration column. The proteins were concentrated to about 10 mg ml^−1^ in a buffer containing 10 mM Tris pH 8.0, 500 mM NaCl and 1 mM DTT. SeMet-labelled protein was expressed in Met-free medium supplemented with 100 mg l^−1^ SeMet and was purified as described above.

### Crystallization and structure determination

Crystals of *Oc*NOD2ΔCARDΔloop (form 1 and form 2) were grown at 4 °C by mixing the protein solution with equal volume of reservoir solution (form 1: 5% PEG20000, 5% MPD, 100 mM Hepes pH 7.8 and 500 mM NaCl, form 2: 5% PEG10000, 100 mM Hepes pH 7.8 and 500 mM NaCl ) by the sitting drop vapour diffusion method. Crystals of *Oc*NOD2ΔCARD^SER^ were obtained under conditions similar to those for *Oc*NOD2ΔCARDΔloop form 1 crystals.

X-ray diffraction data were collected at 100 K on beamlines BL32XU and BL41XU at SPring-8 (Hyogo, Japan). Crystals were equilibrated in a cryoprotectant solution consisting of reservoir solution supplemented with 25% ethylene glycol before flash-cooling. Heavy atom derivatives were obtained by soaking the *Oc*NOD2ΔCARDΔloop form 1 crystals in the reservoir solution supplemented with 5 mM KAu(CN)_2_ or 5 mM K_2_PtCl_4_ for 15 min. The SeMet-labelled protein was crystallized under conditions similar to those for *Oc*NOD2ΔCARDΔloop form 1 crystals.

X-ray diffraction data were processed with HKL2000 (ref. 47). The initial phase for *Oc*NOD2ΔCARDΔloop form 1 crystals was obtained to 2.5 Å by multiple isomorphous replacement with anomalous scattering using phenix.autosol^48^. Several rounds of manual model building using COOT^49^ and structural refinement using Refmac^50^ were performed. The crystal structures of *Oc*NOD2ΔCARDΔloop (form 2) and *Oc*NOD2ΔCARD^SER^ were solved by molecular replacement using Molrep^51^ with the refined model of *Oc*NOD2ΔCARDΔloop (form 1). The quality of the final structure was evaluated with MolProbity^52^. In the structures of *Oc*NOD2ΔCARDΔloop (form 1), *Oc*NOD2ΔCARDΔloop (form 2), and *Oc*NOD2ΔCARD^SER^, 99, 98 and 98% of the residues were in Ramachandran favoured or allowed regions, respectively. The statistics of data collection and refinement are summarized in Table 1. The structural figures were prepared using CCP4mg (ref. 53).

The coordinate and structure factor data of OcNOD2ΔCARDΔloop (form 1), OcNOD2ΔCARDΔloop (form 2) and OcNOD2ΔCARDSER have been deposited to the Protein Data Bank (PDB) under the PDB IDs 5IRN, 5IRM and 5IRL, respectively.

### NF-κB-dependent luciferase reporter assay

HEK293T cells from human embryonic kidney, which were kindly provided by Professor T. Kaisho (Wakayama Medical University, Japan), were cultured in DMEM (Gibco) with 10% fetal bovine serum, 1 × penicillin-streptomycin-glutamine (Life Technologies) and 50 μM 2-ME. To check the constitutive activity of various human NOD2 mutants, 2 × 10^5^ HEK293T cells were seeded in collagen-coated 24-well plates and transiently transfected with wild-type or mutant human NOD2 cDNAs in pFLAG-CMV2 (400 ng) together with pELAM1-luc reporter plasmid (50 ng), using Polyethylenimine ‘Max' (Polysciences, Inc). To check the response to NOD2 ligands by various NOD2 mutants, 2 × 10^5^ HEK293T cells were seeded in collagen-coated 24-well plates and transiently transfected with wild-type or mutant rabbit/human NOD2 cDNAs in pMX-puro-IRES-rat CD2 (10 ng), together with Human SLC15A3 cDNA in pcDNA3.1(−) vector (200 ng) and pELAM1-luc reporter plasmid (1 ng), using Polyethylenimine ‘Max'. At 30 h post-transfection, transfected cells were re-seeded in collagen-coated flat 96-well plates (Corning) at a density of 5 × 10^4^ cells per well and subjected to luciferase assay using the Luciferase Assay System (Promega). Samples for checking response to NOD2 ligands were stimulated with MDP or L18-MDP from Invivogen for 4.5 h and then subjected to luciferase assay. The relative light unit (RLU) of chemiluminescence was measured using a GloMax Explorer (Promega) or a MiniLumat LB9506 luminometer (Berthold).

### Data availability

The coordinate and structure factor data of OcNOD2ΔCARDΔloop (form 1), OcNOD2ΔCARDΔloop (form 2) and OcNOD2ΔCARDSER have been deposited in the Protein Data Bank (PDB) with the accession codes, 5IRN, 5IRM and 5IRL, respectively. The data that support the findings of this study are available from the corresponding author on request.

## Additional information

**How to cite this article:** Maekawa, S. *et al*. Crystal structure of NOD2 and its implications in human disease. *Nat. Commun.* 7:11813 doi: 10.1038/ncomms11813 (2016).

## Supplementary Material

Supplementary Information Supplementary Figures 1-7 and Supplementary Table 1

## Figures and Tables

**Table 1:** Data collection and refinement statistics.

	***Oc*****NOD2 ΔCARDΔloop (form 1)**				***Oc*****NOD2 ΔCARDΔloop(form 2)**	***Oc*****NOD2 ΔCARD**^SER^
	**Native**	**KAu(CN)**_**2**_	**K**_**2**_**PtCl**_**4**_	**SeMet**		
*Data collection*						
X-ray source	SPring-8 BL41XU	SPring-8 BL32XU	SPring-8 BL32XU	SPring-8 BL32XU	SPring-8 BL32XU	SPring-8 BL32XU
Space group	*P*2_1_2_1_2_1_	*P*2_1_2_1_2_1_	*P*2_1_2_1_2_1_	*P*2_1_2_1_2_1_	*P*2_1_2_1_2_1_	*P*2_1_2_1_2_1_
Cell dimensions						
*a*, *b*, *c* (Å)	77.8, 106.2, 185.4	77.2, 108.7, 186.1	78.1, 107.7, 185.4	77.8, 107.2, 185.7	112.2, 122.9, 177.6	77.9, 107.8, 185.3
Resolution (Å)	2.3 (2.30–2.34)*	2.5 (2.50–2.54)	2.9 (2.90–2.95)	2.69 (2.69–2.74)	3.3 (3.3–3.36)	3.1 (3.1–3.15)
*R*_merge_ (%)	5.5 (49.3)	17.5 (86.7)	11.6 (91.1)	0.141 (0.752)	16.4 (71.2)	11.6 (63.2)
*I*/σ*I*	29.7 (2.0)	5.4 (1.2)	23.1 (1.9)	20.4 (1.3)	8.4 (1.6)	12.7 (1.9)
Completeness (%)	92.6 (77.0)	97.2 (81.7)	99.8 (97.0)	85.3 (11.7)	100.0 (100.0)	98.8 (96.2)
Redundancy	6.7 (5.9)	6.7 (5.4)	7.3 (6.9)	9.8 (4.7)	6.1 (6.2)	4.1 (4.0)
						
Refinement						
Resolution (Å)	92.7–2.3	—	—	—	94.8–3.3	93.2–3.1
No. reflections	57,509	—	—	—	35,238	27,203
*R*_work_/*R*_free_ (%)	20.2/23.6	—	—	—	19.3/23.7	19.9/25.0
No.atoms						
Protein	5,833	—	—	—	11,987	5,792
ADP	27	—	—	—	54	27
Oher	47	—	—	—		5
*B*-factors (Å^2^)						
Protein	77.8	—	—	—	80.1	69.7
ADP	55.5	—	—	—	50.8	43.9
Other	61.6	—	—	—		52.8
R.m.s.d						
Bond length (Å)	0.017	—	—	—	0.007	0.009
Bond angles (°)	1.93	—	—	—	1.28	0.981

^*^Highest-resolution shell is shown in parenthesis. X-ray data were collected from single crystals.
