# Supplementary material for: Crystal structure of NOD2 and its implications in human disease
Source: Nat Commun. 2016 Jun 10;7:11813. doi: 10.1038/ncomms11813 (PMC4906405; doi:10.1038/ncomms11813)
Supplement: Supplementary Information — Supplementary Figures 1-7 and Supplementary Table 1 [file ncomms11813-s1.pdf]

Supplementary Figure 1

a

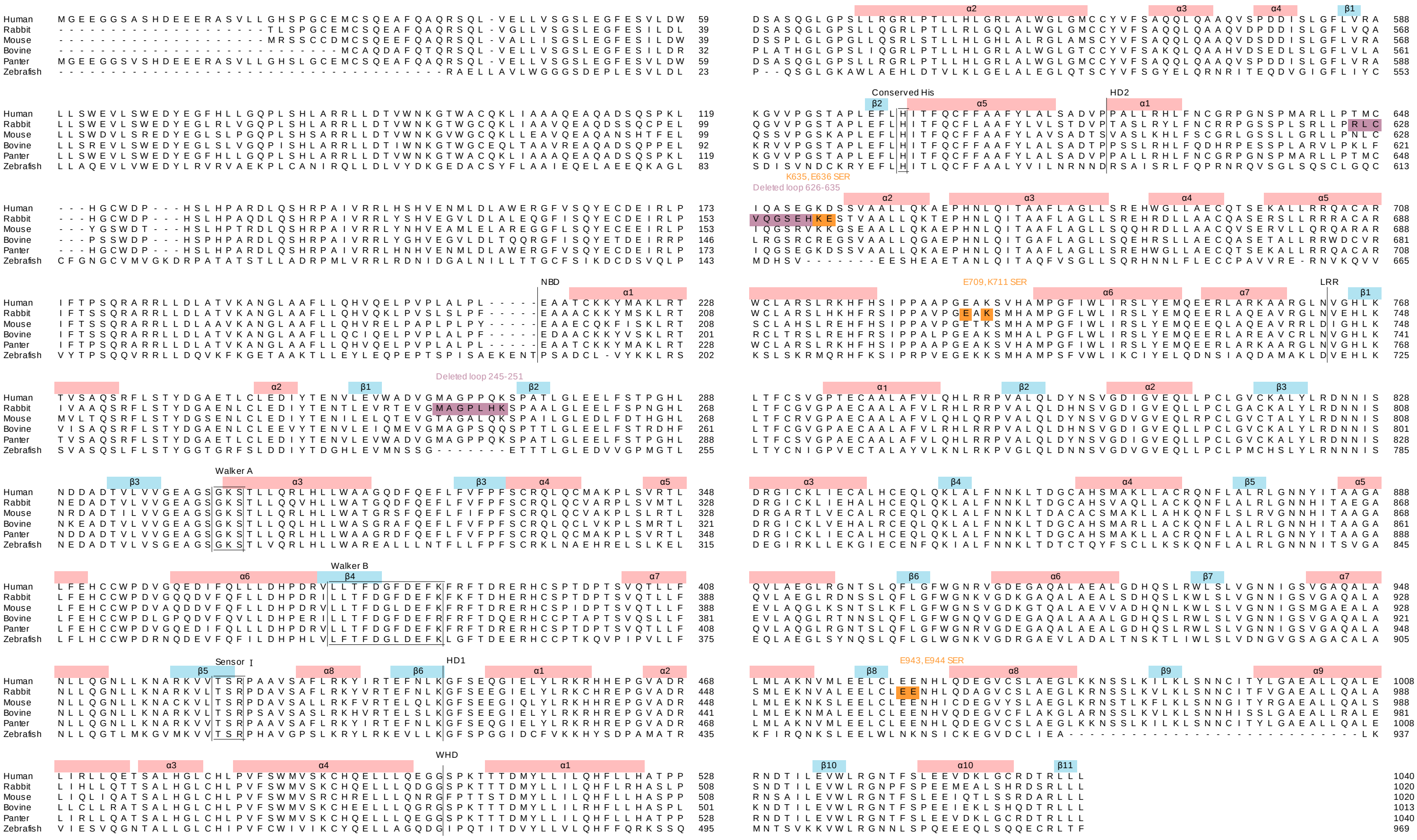

Supplementary Figure 1 (continued)

b

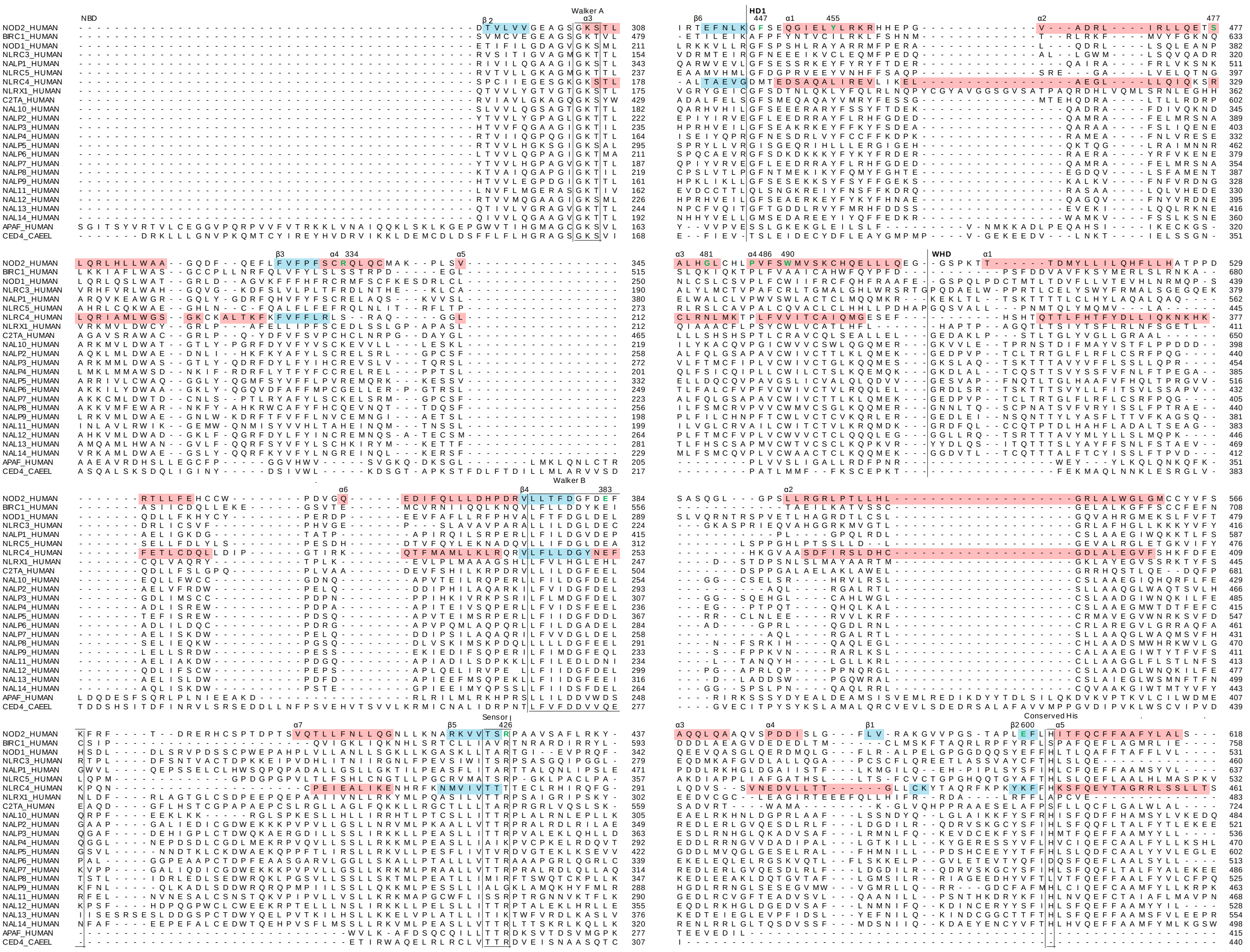

Supplementary Figure 1 (continued)

c

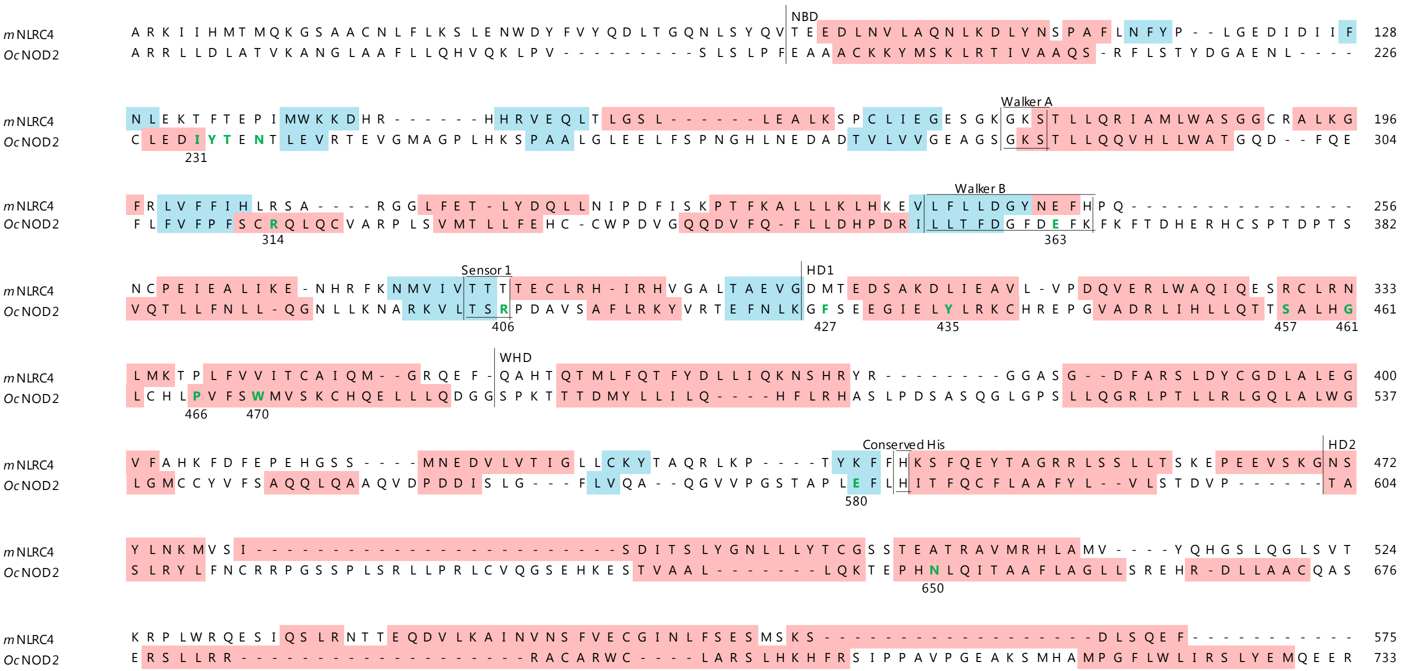

Supplementary Figure 1. Sequence alignment of NOD2

(**a**) Sequence alignment of NOD2 from rabbit (G1T469), human (Q9HC29), panther (Q53B87), bovine (Q6E804), mouse (Q8K3Z0), and zebrafish (F8W3K2). Values in parentheses are Uniprot accession numbers. Secondary structure elements are labeled based on the *Oc*NOD2ΔCARDΔloop form 2 crystal structure. The Walker A, Walker B, Sensor I motifs and conserved His are highlighted in light blue. The deleted loops and SER mutations are highlighted in pink and purple, respectively. Alignments were performed using Clustal Omega software (EMBL-European Bioinformatics Institute).

(**b**) Sequence alignment of NOD2 and other NLRs in NOD domain using Clustal W. α-helices and β-strands are colored in pink and light blue, respectively.

(**c**) Structure based sequence alignment of *Oc*NOD2 and mNLRC4 in NOD domain using MAFFTash (https://sysimm.ifrec.osaka-u.ac.jp/MAFFTash/). α-helices and β-strands are colored in pink and light blue, respectively.

Supplementary Figure 2

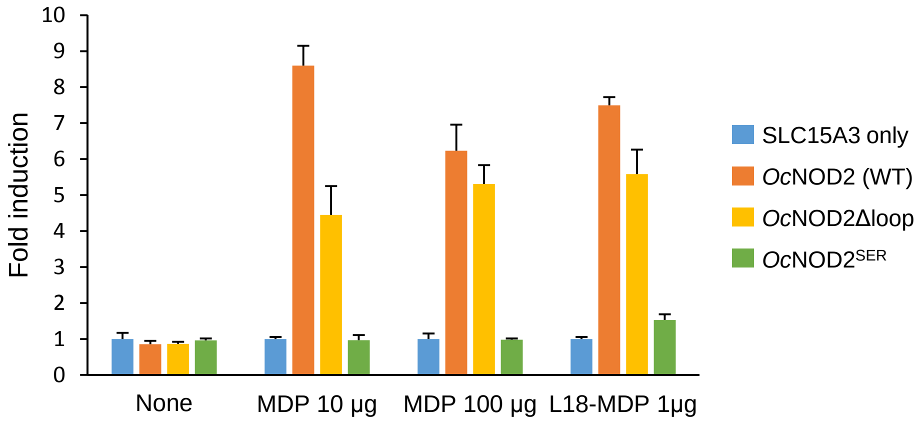

Supplementary Figure 2. NF-κB activation assays

*Oc*NOD2 responses to MDP or L18-MDP.

NF-κB activation of wild-type *Oc*NOD2, OcNOD2Δloop, and OcNOD2^{SER} induced by MDP. Luciferase activity was measured by NF-κB–dependent luciferase reporter assay using HEK293T cells co-expressing rabbit NOD2 and human SLC15A3. MDP was used at 10 µg and 1 µg, and L18-MDP was used at 1 µg. Data represent the mean fold induction of NF-κB activity (n=3, +SD).

Supplementary Figure 3

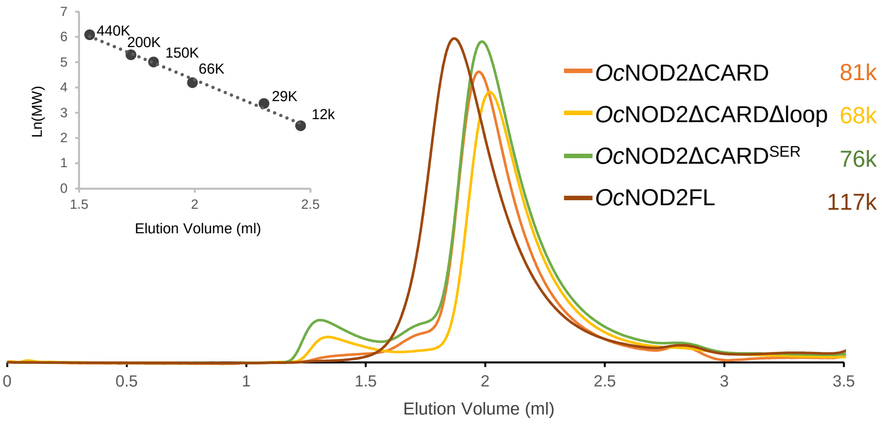

Supplementary Figure 3. Gel filtration chromatography of recombinant *Oc*NOD2

Gel filtration chromatography of *Oc*NOD2ΔCARD, *Oc*NOD2ΔCARDΔloop, *Oc*NOD2ΔCARD^{SER}, and full-length *Oc*NOD2 was conducted using Superdex 200 Increase 5/150 GL column (GE Healthcare) with running buffer containing 10 mM Tris-HCl (pH 8.0), 0.5 M NaCl, 10% glycerol, and 1 mM DTT. The molecular weight of NOD2 proteins was calculated based on the standard curve generated by elution volumes of molecular weight standards: apoferritin (440 kDa), β-amylase (200 kDa), alcohol dehydrogenase (150 kDa), bovine serum albumin (67 kDa), carbonic anhydrase (29 kDa), and cytochrome c (12 kDa).

Supplementary Figure 4

a

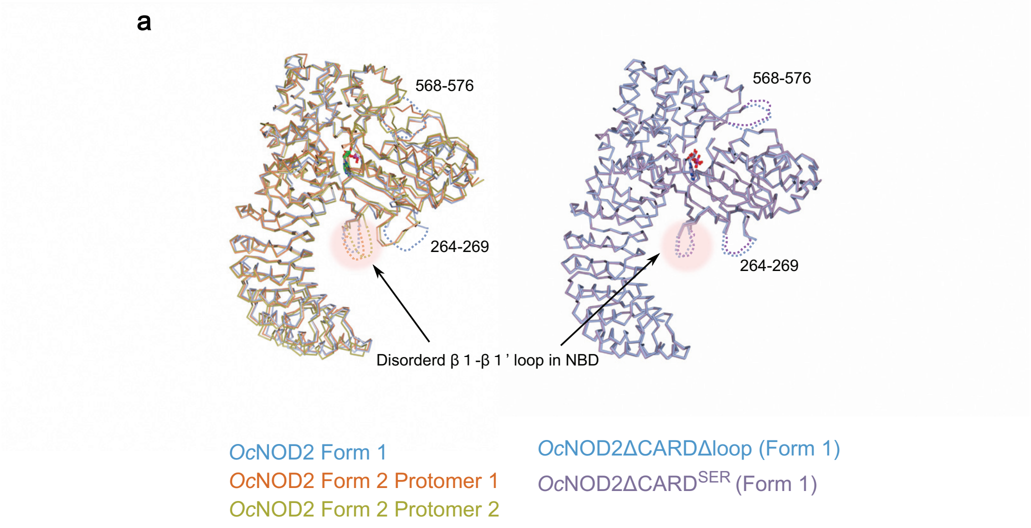

b

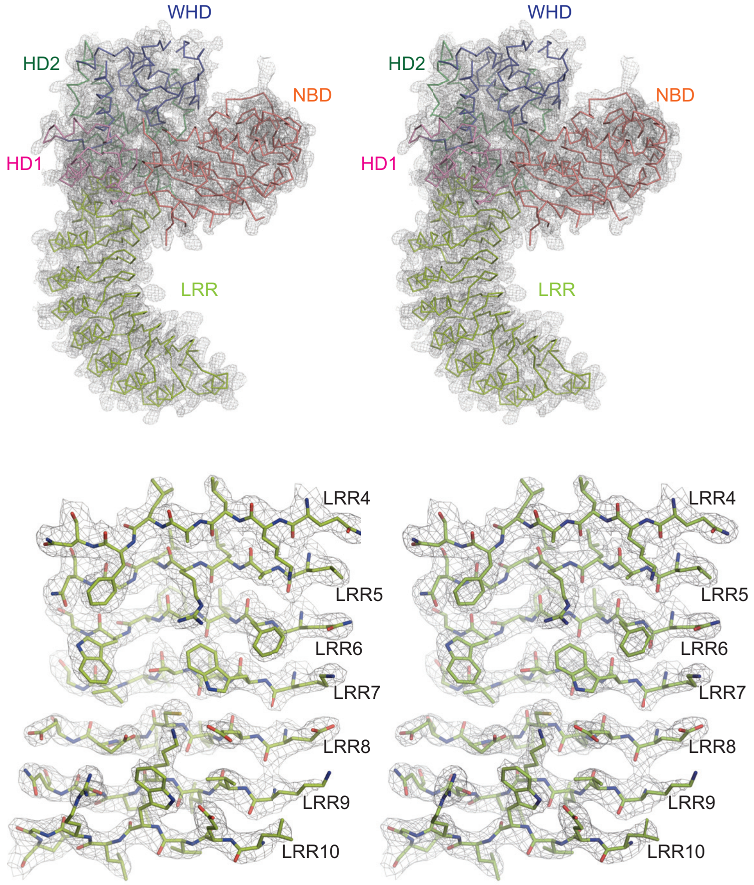

Supplementary Figure 4. Superposition of *Oc*NOD2 structures from different crystals

(**a**) left, Superpositions of *Oc*NOD2ΔCARDΔloop form 1 (cyan), *Oc*NOD2ΔCARDΔloop form 2 protomer 1 (coral), and *Oc*NOD2ΔCARDΔloop form 2 protomer 2 (yellow). The regions missing in the refined model are indicated by dashed lines. right, Superpositions of *Oc*NOD2ΔCARDΔloop form 1 (cyan) and *Oc*NOD2ΔCARDSER (lilac).

(**b**) Stereoview of the electron densities of *Oc*NOD2 (form 1 crystal).

The densities were contoured for the overall architecture (top) and LRR region (bottom) at the 1.0σ and 1.5σ levels in the 2*F*o-*F*c map, respectively.

Supplementary figure 5

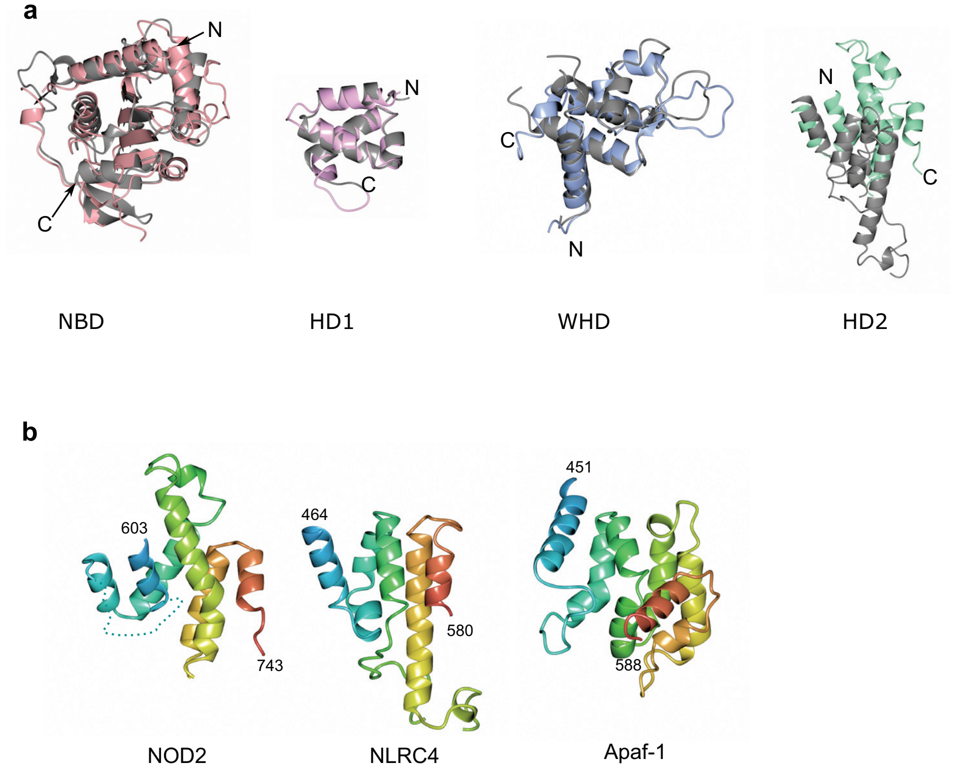

Supplementary Figure 5. Comparison of subdomain structures

(**a**) Each NOD subdomain of *Oc*NOD2 is superimposed with the corresponding domain of NLRC4 (gray).

(**b**) Comparison of the HD2 subdomains of OcNOD2, NLRC4, and Apaf-1. The N- and C-termini are indicated by their residue numbers.

Supplementary figure 6

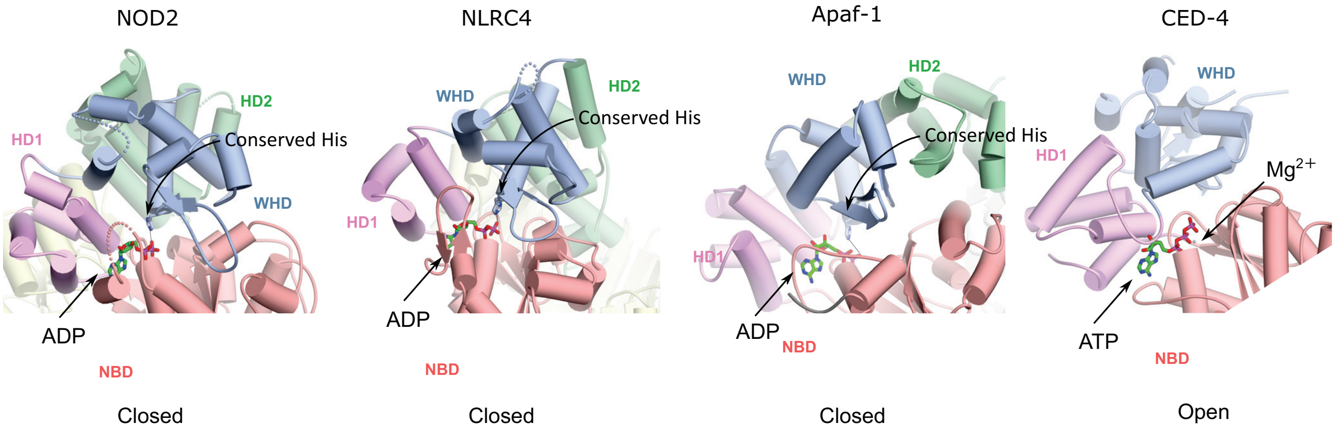

Supplementary Figure 6. Coordination modes of ADP or ATP molecules in the NOD domains

The ADP- or ATP-bound NOD domains of OcNOD2, mNLRC4 (PDB code 4KXF), Apaf-1 (PDB code 3SFZ), and CED4 (PDB code 2A5Y) are shown in similar orientations. The conserved His was drawn.

Supplementary Figure 7

a

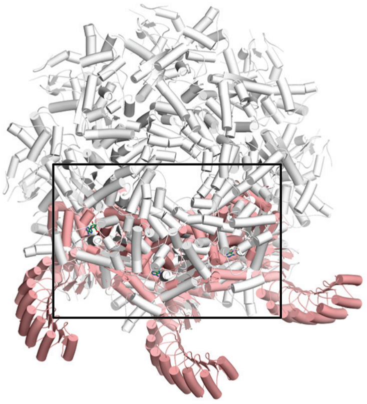

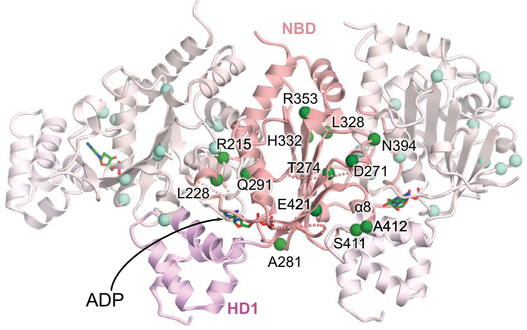

CED-4 apoptosome

b

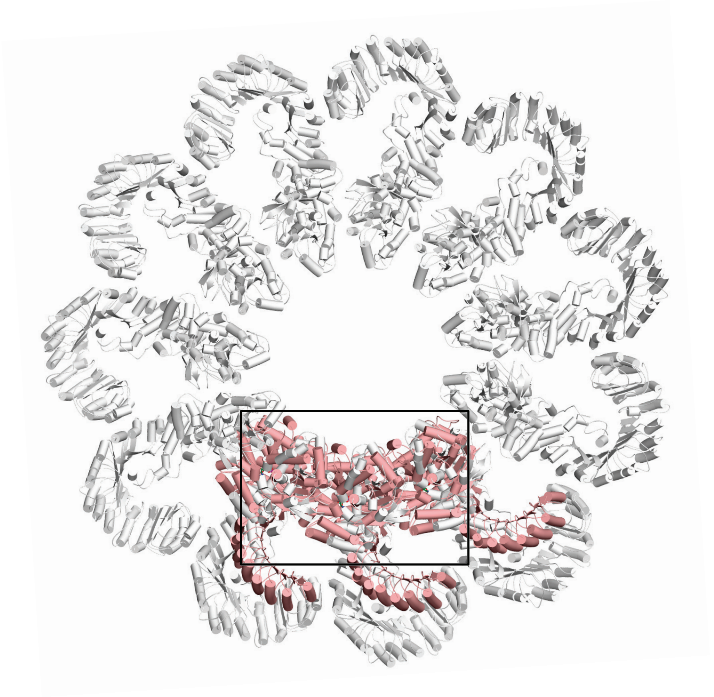

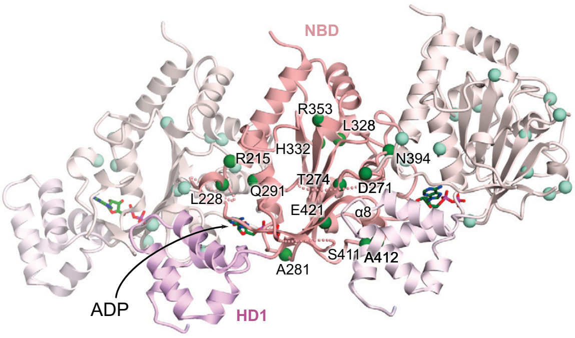

NLRC4 inflammasome

Supplementary Figure 7. Putative oligomeric model of NOD2

The model was constructed by superimposing the *Oc*NOD2 structure onto the CED4 octamers (PDB code 3LQQ) (**a**) and NLRC4-NAIP2 inflammasome (PDB code 3JBL) (**b**) using the NBD domain as reference. NOD2 is colored in pink and other molecules are colored in grey in the left panel. The three adjacent protomers of NOD2 are shown in a magnified view in the right panel. The middle protomer is colored in pink (NBD) and purple (HD1) and the rest of the protomers are colored in light colors. CD-related mutations in the NBD domain are shown as green spheres in the middle protomer and as lightgreen spheres in the adjacent protomers. No severe steric clash would occur in the assembly of NOD2 when we superposed two molecules of *Oc*NOD2 with both protomers of a lateral CED-4 dimers. Superposition with active NLRC4 with a higher order of oligomerization (11mer) resulted in a much closer distance between the two LRR domains of a lateral NOD2 dimer, but LRR domains of two adjacent NOD2 molecules did not clash each other.

Supplementary Table 1 Disease related mutations

Domain	Disease phenotype	Mutation Human (Rabbit)	Location	ASA(Å^2)	Importance in NOD2 and proposed effect of mutations	Disease phenotype	Mutation Human (Rabbit)	Location	ASA(Å^2)	Importance in NOD2 and proposed effect of mutations
NBD	CD	R235C (R215)	Surface	110		BS/EOS	R334Q (R314)	Surface	36	H-bond to E363 and E580
	CD	L248R (L228)	Intra	0	Totally buried	BS/EOS	R334W (R314)	Surface	36	
	CD	D291N (D271)	Surface	112		EOS	D382E (D362)	Intra	13	H-bond to S405, R406 and E363
	CD	T294S (T274)	Intra	4	H-bond to T417	BS	E383G (E363)	Surface	17	H-bond to R314
	CD	A301V (A281)	Intra	12		BS/EOS	E383K (E363)	Surface	17	
	CD	R311W (Q291)	Surface	19	Not conserved (Gln in rabbit)					
	CD	L348V (L328)	Intra	4						
	CD	H352R (H332)	Surface	75						
	CD	R373C (R353)	Surface	81						
	CD	N414S (N394)	Surface	39	H-bond to N389, Q392 and N394					
	CD	S431L (S411)	Surface	60	H-bond to S411 and A412					
	CD	A432V (A412)	Surface	87						
	CD	E441K (E421)	Surface	36	H-bond to R415					
HD1						BS	G464W (G444)	Surface	34	
						BS/EOS	L469F (L449)	Intra	1	No room for Phe
						BS/AML[a]	R471C (H451)	Surface	116	Not conserved (His in rabbit)
						BS/EOS	G481D (G461)	Intra	3	No room for Asp
						BS/EOS	W490L (W470)	Surface	52	Interact with ADP
						BS/EOS	C495Y (C475)	Intra	0	No room for Tyr
						EOS	H496L (H476)	Surface	62	
WHD	CD	A612V (A592)	Intra	0	No room for Val	EOS	M513T (M493)	Intra	0	
						BS	T605N (T585)	Intra	10	H-bond to H583, Y494 and F586
						EOS	T605P (T585)	Intra	10	
						EOS	N670K (N650)	Intra	2	H-bond to R406,H649 and N650
HD2	CD	R684W (R664)	Surface	138	H-bond to R667					
	CD	R702W (R682)	Surface	133						
	CD/UC[b]	R703C (R683)	Intra	7	H bond to V601, S593, L595 and L597					
	CD	R713C (R693)	Surface	99						
	CD	A725G (A705)			Disordered					
	CD/UC	A755V (A735)	Intra	1	No room for Val					
	CD	A758V (A738)	Intra	0						
LRR	CD	E778K (E758)	Surface	30	H-bond to G755, G753 and R724					
	CD	V793M (V773)	Intra	1	No room for Met					
	CD	E843K (E823)	Surface	127	H-bond to H821 and H823					
	CD	N853S (N833)	Intra	9	H-bond to N805, N806, 807, L830, F831, and N833					
	CD	M863V (V843)	Intra	1	Not conserved (Val in rabbit)					
	UD	A885T (A865)	Surface	24						
	CD	G908R (G888)	Surface	21						
	CD	A918D (A898)	Surface	22						
	CD	G924D (S904)	Surface	26	not conserved (Ser in rabbit)					
	CD	V955I (V935)	Surface	118						

[a]AML: Acute Myeloid Leukemia [b]UC: Ulcerative Colitis
